# Education for just transitions: Lifelong learning and the 30th anniversary Human Development Report

**DOI:** 10.1007/s11159-021-09914-w

**Published:** 2021-08-24

**Authors:** Simon McGrath, Séverine Deneulin

**Affiliations:** 1 School of Education, University of Nottingham, Nottingham, UK; 2 Laudato Si’ Research Institute, Campion Hall, Oxford, UK

**Keywords:** Lifelong learning, Just transitions, Human Development Reports, Faith-based learning

## Abstract

The 30th anniversary Human Development Report, entitled *The Next Frontier: Human Development and the Anthropocene,* was released by the United Nations Development Programme in December 2020. It marks an important step forward as a high-profile publication trying to radically re-think the challenge of sustainable development and revisit what it means to develop as human beings interconnected within earth systems. This article provides a critical reading of the report, and human development literature more widely, in assessing the role of lifelong learning in educating for *just transitions,* which it broadly understands as the transformation of all social systems, including economic systems, to bring them back into balance with earth systems in which they are embedded. The report maintains its trademark “human development lens” which has characterised the series since their inception in 1990. It prioritises consideration of capabilities, agency and values as central to the challenge, and opens up a discussion of how we need to change our understandings, values and actions, including what it means to be human, in order to effect just transitions towards sustainability. However, as the authors demonstrate, the report falls short of considering the lifelong learning challenge inherent and central to just transitions. The authors argue that the pressing challenge of responding to the climate emergency requires a richer understanding of how humans learn throughout their life course. In so doing, this article is a contribution to both the literature on education and human development, and the growing body of literature in the field of adult education and sustainability.

## Résumé

L’éducation pour des transitions justes : l’apprentissage tout au long de la vie et l’édition qui marque le 30e anniversaire du Rapport sur le développement – L’édition du Rapport sur le développement, parue à l’occasion de son 30e anniversaire et intitulée La prochaine frontière : le développement humain et l’Anthropocène, a été publiée par le Programme de développement des Nations Unies en décembre 2020. Publication de haut niveau, le rapport constitue une importante avancée dans la tentative de repenser radicalement le défi que pose le développement durable et de revoir ce que veut dire notre développement en tant qu’êtres humains liés entre eux au sein des systèmes de la Terre. Cet article propose une lecture critique du rapport et, plus globalement, de la littérature sur le développement humain, en évaluant le rôle de l’apprentissage tout au long de la vie dans l’éducation pour des transitions justes, qu’il envisage plus largement comme la transformation de tous les systèmes sociaux, y compris des systèmes économiques, afin de restaurer un équilibre entre eux et les systèmes de la Terre dans lesquels ils sont ancrés. Le rapport continue de passer par « le prisme du développement humain », la marque de fabrique qui le caractérise depuis sa création en 1990. Il donne la priorité à la prise en compte des capacités, de l'instrumentalité et des valeurs qui sont au cœur du défi, et ouvre le débat sur la façon dont nous devons modifier nos conceptions, valeurs et actions, y compris en ce qui concerne la signification de ce qu’est être humain, pour mener à bien des transitions justes sur la voie du développement durable. Néanmoins, les auteurs démontrent que le rapport est loin de considérer que le défi de l’apprentissage tout au long de la vie est inhérent à des transitions justes et fondamental pour elles. Ils soutiennent que l’urgence de répondre au changement climatique demande une conception plus riche de la façon dont les humains apprennent tout au long de leur existence. Ce faisant, l’article contribue à enrichir la littérature sur l’éducation et le développement ainsi que celle de plus en plus abondante sur l’éducation des adultes et le développement durable.

## Introduction

The 30th anniversary Human Development Report, entitled *The Next Frontier: Human Development and the Anthropocene* (henceforth referred to as HDR 30), was released in December 2020. It marks a new turn in the human development journey on which the global community embarked with the publication of the first HDR in 1990.

Scientists generally believe that we are exiting the Holocene, which spanned some 12,000 years, during which human civilization as we know it came to be. They propose that we are now entering a new geologic epoch – the Anthropocene – in which humans are a dominant force shaping the future of the planet (UNDP 2020, p. 4).

Given the current context of increased climate emergency in combination with the global pandemic which has laid bare the deep interconnections between all life systems, HDR 30 frames a new human development narrative that sees “social and natural systems as embedded in each other” (ibid., p. 23). This integration of human and earth systems departs from previous reports in that the environment is no longer seen as a separate realm, acting as a constraint upon or facilitator of human flourishing. Humans are part of nature and not separate from it. The human development challenge is therefore “to redress both social and planetary imbalances” (ibid., p. 22)

Although the HDR series, and its subsequent “human development” school of thought, began in 1990, the intellectual underpinnings can be dated back further to critiques of “big push development”^1^ that coalesced in the early 1970s around “basic needs”. As noted elsewhere (McGrath 2018), education was an important element of this account. Contemporary debates in international education, and particularly the influence of Paulo Freire and Ivan Illich, prompted a sense of urgency to think of basic needs achievement in terms of a strong lifelong learning perspective, through a focus on non-formal education (Coombs and Ahmed 1974).

However, by the time the Human Development Index (HDI)^2^ emerged in the first HDR in 1990, schooling had been made a central indicator, and the much trickier lifelong and non-formal dimensions were missing. As we argue in the course of this article, this omission of a lifelong learning perspective in human development literature continues to this day. Despite HDR 30’s attempt to cross a new frontier in human development thought by locating the development of human beings within planetary systems, this absence of a lifelong perspective on education for human development is a significant shortcoming in an age characterised by climate emergency and biodiversity loss. Moreover, it disregards the explicit wording chosen half a decade ago in the United Nations 2030 Agenda. Among the Agenda’s 17 Sustainable Development Goals, the fourth one (SDG 4) aims to

ensure inclusive and equitable quality education and promote *lifelong learning opportunities* for all (UN 2015, p. 19; emphasis added).

The purpose of this article is to address the absence of a lifelong learning perspective in HDR 30. We explore which approaches to lifelong learning could support individuals and communities in the transformation of social, economic, cultural and political systems. We look at a particularly neglected aspect of lifelong learning, namely, the role of faith communities in forming communities of practice (Wenger 1998) and social movement learning (Holst 2018; Walters and von Kotze 2019; Kuk and Tarlau 2020). We critically examine these non-formal adult educational spaces in preparing individuals for just transitions. By *just transitions,* we understand the multiple and cross-sectoral processes of systemic change that are needed to transform current socio-economic and cultural systems into ones which achieve greater balance between social and earth systems while promoting the flourishing of all, rather than merely a few at the expense of others (Swilling and Annecke 2012; Swilling 2020; Newell and Simms 2020; Renouard et al. 2021) . We explore the role of social movements in lifelong education and learning, and of faith communities therein, for “a just transformation that expands human freedoms while easing planetary pressures” (UNDP 2020, p. 9).

## HDR 30 and education

As suggested by its title, *The Next Frontier: Human Development and the Anthropocene,* HDR 30 addresses the challenge of living in the Anthropocene and its implications for thinking about development and social change. Notwithstanding critiques of the problematic nature of the Anthropocene concept (e.g. Moore 2016), this approach marks an important step forward for such a prominent international development publication in its engagement with the climate emergency. As the report notes, from the human development perspective, economic development was always only understood as a means to the end of human flourishing. But HDR 30 now frames human flourishing within the flourishing of all ecosystems. It contends that “the human development journey cannot be separated from the web of life we are embedded in” (UNDP 2020, p. 21). It urges for the human development journey to be re-imagined, not only in putting people first, but in putting “people embedded in the biosphere” first (UNDP 2020, p. 223).

### Human agency and choices

One of HDR 30’s innovations, though in keeping with the HDR tradition, is its dealing with development in the age of the Anthropocene through a focus on human agency and choices. It characterises the Anthropocene as an “age defined by human choice” (UNDP 2020, p. iii). If we have, through our actions, modified Earth’s crust, so too we can now choose to act differently to address current social and planetary imbalances. Like the Intergovernmental Panel on Climate Change (IPCC) *Special Report on the Impacts of Global Warming* (Masson-Delmotte et al. 2018) and the Intergovernmental Science-Policy Platform on Biodiversity and Ecosystem Services (IPBES) *Global Assessment Report on Biodiversity and Ecosystem Services* (Brondizio et al. 2019), HDR 30 issues a stark warning in terms of the fatal consequences of making the wrong choice: choosing to continue business as usual risks leading planet Earth into a sixth mass extinction. The report urges us to make different choices, both at individual and collective levels.

It is a particular way of exercising our human freedom that has led to the current imbalances. As HDR 30 puts it, “Human choices, shaped by values and institutions, have given rise to the interconnected planetary and social imbalances we face” (UNDP 2020, p. 5). As noted elsewhere, HDR 30

makes several references to [Amartya] Sen’s argument that “[t]he reach of reasoned and interactive agency […] can be particularly crucial for our transition to sustainability” (Sen 2013, p. 18). It discusses the role of public reasoning, agency, and collective action in changing a society’s values around nature and social norms about what is acceptable or non-acceptable behaviour – for example, flying to a destination when less carbon-intensive means of transport are available (Deneulin 2021, p. 100),

or eating less meat or walking or cycling whenever possible (UNDP 2020, p. 143). HDR 30 remains silent, however, on the power of corporations to shape people’s behaviours through the advertising industry beyond reference to some experiments on consumer responses to green advertising. The key question the report asks is: “How can we use our power to expand human freedoms while easing planetary pressures”? (UNDP 2020, p. 70).

### Capabilities, agency and values

In order to exercise our agency towards just transitions, HDR 30 argues, we need to understand that capabilities, agency and values are inseparable. The report notes that the expansion of capabilities *can* result in pressure on planetary boundaries in countries that are already heavy users of finite resources, and that one cannot simply assume that people will make the right choices. As HDR 30 puts it:

Nor can we simply assume that expanding agency on its own means that more empowered people will invariably choose, individually and collectively, to avoid dangerous planetary change. Values, especially how they stack up and interact, help provide the overall direction for the choices that empowered people make about their lives. Values are fundamental to our personal understanding of what it means to live a good life. But people cannot realise their values without having sufficient capabilities and agency (UNDP 2020, p. 6).

This affirmation is packed with unanswered underlying questions. *Values* are generally thought of as what people hold important in their lives and provide a horizon to orient their priorities and their decisions (Schwartz 2012). But what makes people prioritise certain values over others in their choices? What are the processes through which someone prioritises, for example, the value of perceived security through buying a sport utility vehicle (SUV) over the value of solidarity with those suffering from climate change through buying a car that emits less carbon dioxide or choosing public transport? What does it mean for people to “realise their values”? Does it refer here to the importance of collective action and sufficient political voice and argumentative and organisational skills? In that regard, HDR 30 notes that

Many vulnerable communities lack the financial resources and organisational clout to sustain a long-term fight when there is a threat to their wellbeing. […] When they try to speak out and defend their lives, they are limited […] by asymmetries in power that muffle their voices (UNDP 2020, pp. 67–68).

Or does the statement about realising values refer to financial and other constraints that people face in their abilities to choose sustainable lifestyles or meaningful employment that will contribute to just transitions? What we mean here, for instance, are employment opportunities in the renewable energy sector or widespread opportunities for consumers to buy plastic-free goods.

HDR 30 tackles the relationship between capabilities, agency and values in its chapter 4 entitled “Empowering people, unleashing transformation”, which, according to its summary statement, “emphasises the importance of education and identifies ways in which catalytic action can ripple across society, helping to shift norms and empower people to act on their values” (UNDP 2020, p. 132).

After conceding that values and actions are often not well-aligned, the chapter then acknowledges “that education and lifelong learning have contributed to the formation of values that support the idea of stewardship of the planet” (UNDP 2020, p. 133).

### Lifelong learning

Although lifelong learning is implicitly of importance to the human development approach, as noted above, it has not featured strongly in the evolution of an account of education’s role in human development. In part, this seems to be due to the pragmatics of developing a human development index. Data on schooling are far easier to come by than data on adult education, given the myriad and often informal manifestations of the latter. Moreover, the apparent harmony of rights-based and human capital cases for primary education around 1990 made a focus on school-based indicators seem self-evident (McGrath 2018). In the context of the six Education for All goals (including adult education) (UNESCO 1990, WEF 2000) being reduced to two education-related Millennium Development Goals (on schooling only) (UN 2000), the emerging research literature on education and human development came to be dominated by a focus on schooling. Though higher and vocational education strands have since become important, an adult education and human development body of literature has been slower to emerge. With SDG 4 talking about lifelong learning (UN 2015), as mentioned earlier, the moment appears ripe for human development to address this gap.

In the light of this, the above-cited mention of *lifelong learning* in a Human Development Report seems significant. However, HDR 30 quickly moves on (within the same paragraph on p. 133) to suggest that answers to “what can be done to empower people to act on their values” lie in “social psychology and economics literature” (UNDP 2020, p. 133). It is soon apparent that while HDR 30 acknowledges that lifelong learning has a role to play, it holds that lifelong learning *literature* has nothing to contribute to the transition agenda. Indeed, the above quotation is the only occurrence of the term “lifelong” in the entire HDR 30, which has a total of 400+ pages. Is its sole use simply a mirroring of SDG 4 usage rather than any real engagement with the radical implications of the shift in global goals from schooling to lifelong learning?

### Adult learning and education (ALE)

Later in the chapter, there are about two and a half pages on the question “Where do adults learn?” (pp. 139–141). The overall answer is:

the workplace (trainings, seminars), social interaction (including social media) and government communications (such as governmental awareness campaigns or political discourse) (UNDP 2020, p. 139).

These are not wrong, of course, but they do amount to a profoundly limited account that is exclusionary of other important spaces where adults learn, such as adult education centres, communities and social movements. While the adult learning venues and contexts mentioned by HDR 30 do loosely cover the market, the state and the internet (of course, hugely shaped by both of the former), any sense of societal organisations or civil society itself is missing from the HDR team’s conceptualisation. This seems a great lacuna given the critical importance of social movements in lifelong learning and social change. Examples include the Transition Network snd Extinction Rebellion movements in Europe,^3^ the Via Campesina global movement towards agro-ecology,^4^ more contextual social movements such as MST in Brazil,^5^ and faith communities such as the Eco-Congregation initiative of the Church of England and the Laudato Si’ Action Platform of the Roman Catholic Church.^6^ The omission of faith-based civil society actors, and the lifelong learning which occurs within faith communities, is indeed also noteworthy. While HDR 30 does allude to the similarities between its re-imagined human development journey and that of Indigenous cosmologies, Islamic and Christian traditions (UNDP 2020, p. 88), it falls short of even mentioning the transformative potential of these faith communities in shaping people’s values and actions. Yet, every week, hundreds of millions of adults all over the world meet to pray, to listen to and reflect on the foundational texts of their religions and consider what these may imply for the way they live and the choices they make.

Moreover, for all the HDR’s philosophy of open-ended human development, its view of learning is highly instrumental, top-down and Northern-centric. It lauds the “leadership roles that employers assume for their employees” (UNDP 2020, p. 139) with no critical commentary on the extent to which formal employment is a minority experience globally. Despite the leading role that trade unions have played in just transitions work internationally, there is no mention of trade union education in this area, which builds on a much older tradition of workers’ education.

Within the section headed “From learning to value formation”, the comparatively short sub-section on “Where do adults learn?” is followed by “Where do we stand with our values?”. This *could* continue a discussion on adult education, but instead the focus is on social attitudes surveys and neuroscience. Similarly, the subsequent section “From values to self-reinforcing social norms” and its first two subsections “Harnessing agency” and “Unleashing change through policies” proceed without a single reference to the phrase “adult learning” or its synonyms.

### The literature (not) underpinning HDR 30

While the well-respected journal *Environmental Education Research* gets 14 references in the report’s chapter on education (largely for work on schools), there is not a single reference to a leading international journal in lifelong or adult learning and education. We know from experience that writing reports of this kind with a tight deadline involves a frantic process of rapid gathering of information from fields with which the team are often unfamiliar. Nonetheless, it is striking that seminal adult education literature is entirely absent from HDR 30. Is it because, for some reason, no one in the writing team and advisory board thought to look for literature from the fields of adult education/lifelong learning and social movement? Or is it because the team concluded that this literature did not suit their purposes?

Given that the *International Review of Education* (*IRE*) is edited by a UNESCO Institute and produced five special issues featuring the keyword sustainab* in the past five years, this journal’s absence from HDR 30 does appear noteworthy. This significant lacuna in this otherwise ground-breaking report for re-imagining the human development journey in the age of climate and biodiversity emergency begs us to ask the questions: “what might the body of adult education/lifelong learning literature have contributed?”; “what could be the role of alternative education spaces for learning about becoming individuals and communities who live more justly and sustainably?”; or, to paraphrase the “learning to become” motto of the UNESCO Futures of Education initiative,^7^ “what could be the contributions of adult education/lifelong learning for learning to become?”

In passing, we can ask both what this neglect of UNESCO’s work signifies for the long-standing rhetoric of “One UN” (UN 2005) but also what UNESCO and the adult education community might do to build a conversation with the human development community.

## Contributions from adult education/lifelong learning

Unsurprisingly, adult education literature has much to say about “where adults learn”. Engagement with this literature would have provided a useful corrective to the emphasis on state, employers and social media present in chapter 4 of HDR 30. However, even more important than locating learning venues and contexts is the consideration of questions concerning how learning builds through knowledge and understanding, generating values and norms change, and finally action in pursuit of just transitions. Adult education as a field has long seen learning as tool for personal liberation and social justice (Steele 2000), and has long noted that the relationships between education, value change and transformative action are challenging. In this section, we unpack these relationships through three strands of the lifelong learning body of literature: (1) transformative learning; (2) adult learning and sustainability; and (3) Education for Sustainable Development (ESD) and adult education.

### Transformative learning

HDR 30’s concerns about the relationship between values, value change and actions seem to echo a long-standing and important debate in adult education, closely associated with the evolving account of transformative learning by Jack Mezirow (1991) and others, and subsequent critiques and refinements. According to Mezirow, it is important for us as humans to critically reflect on our experiences leading to a new frame of reference. These can then guide our actions. Mezirow’s work has recently been applied to the challenge of post-COVID learning (Eschenbacher and Fleming 2020) precisely because it is directed at learning for radically changed circumstances. This is clearly analogous to the challenge laid out in HDR 30 in moving towards just transitions, and changing our frames of reference – from seeing ourselves as separate from and masters of ecosystems to seeing ourselves as embedded stewards.

Mezirow’s approach has been criticised by many who argue that it is too rational and cognitive (e.g. Dirkx 1997), although Mezirow did engage with such critiques and seek to modify his position. However, he did not embrace some of the critiques’ focus on the transformation of the interior self, that transformative learning involves “deepening our understanding of ourselves, of the inner worlds which seem so much a part of us but yet so distant from the everydayness of our normal, waking lives” (Dirkx et al. 2006, p. 129). John Dirkx’s vision of a transformative adult education is about an education of the soul, explicitly discerning a central spiritual dimension in adult learning. Given that HDR 30 also mentions the importance of paying attention to the “value of people’s inner lives” (UNDP 2020, p. 112) – a point which is increasingly emphasised in sustainability literature (Ives et al. 2020) and development studies literature in the wake of the decolonising movement (Batliwala 2020) – the neglect of adult education in the transformation of people’s inner lives is an important shortcoming that needs to be further addressed by education and development literature.

Another point from Mezirow (1991) which HDR 30 could have borrowed is that, while all learning brings change, change is not necessarily transformational. He follows Freire’s lead in critiquing transmission approaches to learning. This is why transformational learning must be counter-hegemonic (Brookfield 2000). Referring to the COVID-19 pandemic, Saskia Eschenbacher and Ted Fleming point out that “[t]ransformative learning is also politically dangerous, as this crisis reveals structural injustices” (Eschenbacher and Fleming 2020, p. 662)

Within the HDR 30 agenda, the challenge for the notion of transformative education lies in moving from *knowing* to *being* and *doing* in the context of the sustainability imperative. This insight has already led to a sizeable body of literature on transformative learning for sustainable development. In their summary of this corpus, Joanne Moyer et al. (2016) argue for a stronger focus on the learning–action nexus. Their research with environmental groups in Canada and Kenya has led them to the conclusion that action is pivotal to learning because it allows for embodied learning, which they argue is key to learning the skills necessary to address the sustainability challenges (Moyer et al. 2016, p. 323).

In a systematic review of studies that they and their colleagues conducted over 20 years on the relationships between transformative learning theory and environmental change, Joanne Moyer and John Sinclair (2020) reflect on how the transformative learning lens made them initially downplay the “instrumental” aspects of this learning as less meaningful than “transformative” elements. Their recent reanalysis (ibid.) led them to realise that practical learning of knowledge and skills is often foundational to further learning. Moreover, they also saw more clearly how “instrumental” and “communicative” learning interacts. Crucially, they argue that many actions contributing to sustainable change are not driven by learning but by other forces such as legislation, and that the role of collective action is critical in overcoming barriers.

### Adult learning and sustainability

While Moyer and Sinclair draw heavily on transformative learning theory as a way of thinking about adult learning for sustainability, this is only one strand of recent adult education responses to the climate crisis. Indeed, as noted already, the terms sustainable/sustainability feature in the titles of five special issues published by this journal since 2016.^8^

Introducing the 2016 special issue on “Societal sustainability”, Marcella Milana et al. (2016a) reaffirm the centrality of *adult* learning to the SDGs. In particular, they focus on SDG 16: “Promote peaceful and inclusive societies for sustainable development, provide access to justice for all and build effective, accountable and inclusive institutions at all levels” (UN 2015, p. 28). However, in a separate article they contributed to this special issue, they also chart the limitations of adult education policy in responding to this challenge, seeing this response as doubly constrained by the marginalised and domesticated place of adult education in national learning systems and the contradictions inherent in the notion of sustainable development (Milana et al. 2016b; see also Holford 2016). In a later issue of this journal, Silke Schreiber-Barsch and Werner Mauch (2019) stress the need to move away from adult learning and education (ALE) *for* sustainability towards ALE *as* sustainability. By this, they mean that it is not good enough to simply see ALE as a transmission mechanism for “correct” environmental messages, but that ALE must be itself a practice that is democratic, just and sustainable. This mirrors a key argument in recent international education and development literature regarding the need for a double transformation of education as part of just transitions (e.g. McGrath 2020; Tikly 2020).

#### The importance of place

Educational research more generally has increasingly made the point that place, or territorial context, matters. In several adult education traditions, learning is seen as needing to build from the lived experiences of learners in their specific economic, social, political and cultural contexts. Thus, learning about the climate crisis and the need for just transitions begins, in this view, from local experiences of the challenges caused by immediate and local violation of environmental boundaries before working outwards to how these relate to problems with planetary boundaries and their global causes (Wals et al. 2017). Such learning is built on local and collective problem-solving rather than starting with knowledge transmission from above/outside bringing approved environmental messages, as implied in HDR 30.

However, this local focus is inherently linked to a global perspective, as the local environmental crisis necessarily is interlinked with global aspects of the climate crisis. Crucially, in the Global North, the most obvious effects may not be as apparent. Hence, there has been an increase in learning programmes that seek to build Northern awareness of the impact that actions in the Global North can have in the Global South. These harmful actions include, for example, pension funds investment in the fossil fuel industry or in businesses which violate human rights, or the impact of certain forms of consumption on the countries which produce them (e.g. asparagus, avocado) or supply the raw material (e.g. lithium for electric cars in Europe), and ecosystem destruction in Latin America.

One part of this restating of the importance of place is an openness to learning approaches such as field trips and study tours to explore how things are done in other places (e.g. a rewilding project of a disused space in the United Kingdom [UK]) or making and watching documentary films and building connections to people affected by one’s actions (e.g. the impact of one’s avocado consumption on water strain in Chile).^9^

#### The importance of community learning and a dialogic approach

The past decade has also seen a revitalisation of the notion of community learning centres in both the Global South and the Global North (Rasmussen and Staugaard 2016; Rogers 2019). Alan Rogers argues that such non-formal education settings are an ideal organisational and spatial home for lifelong learning as sustainability, although Palle Rasmussen and Hans Jørgen Staugaard (2016) do highlight the challenges in making these work. As we argue below, the considerable potential of faith community-based learning for providing transformative avenues for learning to being and doing for just transitions is also worth exploring.

On top of a growing awareness of the challenges of increased climate emergency, the global pandemic has accelerated the pressing need for action, and both scholars and practitioners have shared their insights and experiences in recent publications. So, what can we learn from adult education reflections on responses to COVID-19? In some ways, the arguments about what needs to be done to respond to the COVID and climate emergencies are similar, not least because the relationship between COVID-19 and the rise of zoonotic diseases on the one hand, and the destruction of animal habitats and biodiversity loss on the other hand, has now been well established (UNEP 2020). Keri Facer et al. argue that

while the provision of clear, accurate and trustworthy information is essential during a pandemic, public education should not be limited to this approach alone. Instead, dialogic and situated community-based forms of public education are needed, premised on two-way communication with communities (Facer et al. 2020, p. 1).

They suggest that such an approach is better equipped to facilitate the move from knowledge to action than what they call the “inform and enforce” model. Moreover, they suggest that these dialogic approaches, influenced by the Freirean tradition, allow for structural inequalities to be surfaced in ways that “hold […] states accountable for practices that sustain inequities” (ibid., p. 2).

These suggestions are radically different from what pertains under the more top-down approach envisaged by HDR 30’s chapter 4. Indeed, this chapter seems to be out of step with the pleas made in HDR 30 “to seriously attend to the structural conditions and violence creating and perpetuating inequalities – and listen to and include the experiences and priorities of those most marginalised” (UNDP 2020, p. 113). Community-based adult education could provide powerful spaces where the experiences of those most marginalised are listened to. This has, for instance, long been how the Fairtrade movement has made actors aware of the structural inequalities surrounding the production of many foodstuffs.^10^

Social learning and activism are central to this approach to learning. It is necessarily relational and cannot simply be about learning. It must always be located within a recurrent process of learning, acting and reviewing. Community learning is situated in community action, such as digging wells, removing refuse from a park or planting a community vegetable garden. The approach seeks, wherever possible, to build from local examples of self-organisation and to respect local knowledge while seeking to bring it into a dialogue with scientific knowledge.

### Education for Sustainable Development (ESD) and adult education

It is clear that the bulk of work on education for sustainable development has focused on the school sector, so it is not surprising to see HDR 30 refer overwhelmingly to school-focused research on ESD. However, this is to ignore the growing prominence of climate-focused social movements in adult education. In the UK alone, we can point to the work of Extinction Rebellion and the Transition Network movement, as well as many others. It is even more striking that one of the *IRE* special issues, “Non-formal and community learning for sustainable development”, guest-edited by Arjen Wals, Alexander Leicht and Yoko Mochizuki (2017), comes out of UNESCO-affiliated work on ESD in adult, community and non-formal education. That some of this was co-written with UNESCO staff makes its neglect in a UNDP report even more noteworthy.

Several themes emerging from that special issue, which emanates a powerful sense of relationality, could have been very useful for HDR 30’s analysis. Heila Lotz-Sisitka et al. (2017, p. 899) summarise the issue’s focus on understanding better “how sustainable transformations emerge from community learning processes”. Many of the contributions are about learning and community, considering both already existing communities now engaged in climate action learning or learning groups brought together for this purpose. Such communities are seen as being located within wider ecosystems of learning, being and doing that are multi-scalar and complex. The special issue highlights that community learning is generally seen as requiring some catalytic agent, but demonstrates that the type of agent can vary from an inspirational local individual at one end of the spectrum to existing community structures at the other.

In another contribution to the same special issue, considering the nature of learning as social, Robert Didham et al. (2017) remind us to think about what enables a community to come together to learn, drawing on communities of practice literature. They suggest five types of enablers:

(1) *belonging,* which is framed by mutual engagement, reflexive exploration and a coordinated alignment of group identity […]; (2) *commonality,* which reinforces the importance of a shared sense of purpose and common interests among a group of participants who engage in reflective practice; (3) *situatedness,* which draws on the concept of situated cognition and posits that learners obtain both implicit and explicit knowledge when learning is embedded in rich social contexts; (4) *infrastructure,* which considers the need to promote and facilitate participation and ensure accountability for the long-term continuation of communities of practice; and (5) *interdependence,* which is established when the various members of a group of learners bring unique skills and expertise, as well as differing demands, to the group (Didham et al. 2017, p. 835; italics in original)

#### Transgressive learning

Several authors in the 2017 special issue (*IRE 63*:6) argue for a notion of transgressive learning. In a later article, published in a special issue on “Transgressive learning and transformations to sustainability” of a different journal, *Sustainability,* Thomas Macintyre et al. describe this as

radical forms of learning-based change for socio-ecological change within the framework of climate change […] a form of learning which encourages transformation and seeks to disrupt norms and structures which maintain an unsustainable status quo (Macintyre et al. 2020, p. 2).

Transgressive learning seeks to get beyond the individualist and cognitive biases of transformative learning, and is suspicious of socialisation both to existing norms and to new norms imposed from outside. Instead, it seeks to be transgressive in its rejection of the status quo. It explicitly challenges areas of what it sees as injustice, such as colonial practices and overconsumption. However, it is not only concerned with the problems and knowledges of the moment, but also seeks to create new forms of knowledge to address emergent problems.

Transgressive learning draws on Yrjö Engeström’s work on expansive learning (e.g. Engeström 2016). His approach is explicitly about “what is not yet there” and how to germinate it, echoing social movement literature on hope, prefiguring new forms of relationships and models of production around the values of solidarity, equality and sustainability that are “not yet” fully established but already present in our midst (Dinerstein and Deneulin 2012; Dinerstein 2015).

#### Social movement learning

Drawing on the popular education tradition (Freire 1972), there is a growing body of literature on social movement learning, and the role that civil society plays in the type of transgressive learning discussed above. One could name here the feminist movement transgressing socially accepted patriarchal norms, the labour movement transgressing capitalist economic relations, the environmental movement transgressing the prevailing culture of consumerism (Walters and von Kotze 2019; Kuk and Tarlau 2020), the Indigenous social movements in Latin America transgressing anthropocentrism and seeking to recognise rights to nature itself (Espinosa 2014) and the agroecology social movement transgressing the domination of capital over nature (Rosset et al. 2019). However, this literature on social movement learning neglects the role of faith communities in popular education, even though more than 80 per cent of the global population is estimated to belong to a religious group.^11^

## Faith communities and education for just transitions

The importance of religion in development has now been well established in international development circles (Tomalin 2013, 2020), and there is a growing recognition of the role of religious actors in environmental social movements (Kidwell 2020). Yet, as we noted above, the role of faith actors is still largely absent from adult education literature, despite the fact that hundreds of millions of adults come together regularly to be inspired by their foundational religious texts for the way they live and the decisions they make. Furthermore, adult education literature also neglects the roots of many social movements in the emancipatory reading of religious texts, as seen in the US-American civil rights movement or the Latin American democratisation movements of the 1960s and 1970s (Streck and Zanini Moretti 2017). Faith communities’ potential contributions are considerable for lifelong, transformational and transgressing learning towards just transitions, and towards promoting human flourishing and transforming economic and social systems to bring them into balance with planetary systems.

In their review of popular education and social movement learning, Shirley Walters and Astrid von Kotze (2019) note that:

Unlike dominant global models of education, popular education is not about identifying skills deficits in order to better prepare individuals for the marketplace but rather, it seeks to […] challenge the individualised, commodified, social world (Walters and von Kotze 2019, p. 8).

They see popular education as a “critical awakening of people to the ideas and forces of power that shape everyday reality in unequal and unjust ways”, and as a vehicle “to help people realise that such change requires a collective struggle” (ibid.). With reference to Freire (1972), they emphasise that

A popular education that acknowledges the connectedness and interdependence of all living things, and the mutual obligation to respect (rather than simply use and exploit) the living and the spirit world is one means – and indeed an end – towards turning the dream into reality (Walters and von Kotze 2019, p. 8).

The centrality of the dream and turning it into a reality in Freire’s work must be located in his context of the liberation theology of the Brazilian Catholic Church of the 1960s and 1970s. Then, the dream included social democratic governments in Latin America, and a world where every person could read, write and stand for her dignity and uphold their inalienable rights as human beings (Streck and Zanini Moretti 2017). The Brazilian Catholic Church is continuing to uphold a dream, not only for its territory, but for the entire world to which it, and especially the Amazon rainforest, is connected.

In October 2019, at the request of the Catholic bishops of the Amazon region, Pope Francis convened a Special Assembly to discuss social and environmental degradation in Amazonia. Delegations of Indigenous peoples, civil society activists, Church leaders and pastoral workers travelled to Rome for one month to attend the Amazon Synod and discuss the situation of the Amazon and how faith communities, in the region and globally, could respond. The binding document for the whole Catholic Church that emerged from the Synod, *Querida Amazonía* [Beloved Amazon] is arranged around four dreams: a social one, a cultural one, an ecological one and an ecclesial one (Francis 2020).

The *social dream* is one where every person in the Amazon forest is able to live in dignity with their rights being respected. This entails developing a critical awareness of our participation in “colonizing interests […] that have expelled or marginalized the indigenous peoples” (Francis 2020, p. 8), such as through investment in businesses which destroy the environment and its people. The *cultural dream* is about respecting and affirming people’s cultures, to stimulate inter-cultural encounters and recognise one’s own participation in colonising structures which have failed to respect the richness of people’s roots. The *ecological dream* is about overcoming the human–nature dichotomy and recognising the interconnectedness of all living beings, and that “the care of people and the care of ecosystems are inseparable” (Francis 2020, p. 42). As for the *ecclesial dream,* it is about the transformation of the Catholic Church itself, and its 1.3 billion members and thousands of institutions worldwide, towards modes of functioning and being that restore the balance between social and earth systems. Each Catholic institution is urged to reflect critically on its own involvement in structures which damage the environment and lead to human rights abuse, such as in their investment portfolio, and what they can change, such as locally and ethically sourced food for school meals, or actions aimed at reducing carbon emissions.

To turn these four dreams into reality, the Catholic Church has developed a global lifelong learning initiative, called the Laudato Si’ Action Platform (see footnote 6). The platform is based on the encyclical letter *Laudato Si’* [Praise be to you]: *On Care for our Common Home* (Francis 2015), issued by Pope Francis in 2015 and addressed to every person on the planet. In this letter, which constitutes the mother document of the Amazon Synod, he

urgently appeal[s] for a new dialogue about how we are shaping the future of our planet. We need a conversation which includes everyone, since the environmental challenge we are undergoing, and its human roots, concern and affect us all (Francis 2015, p. 11).

A similar initiative is being prepared by the global Muslim community, which is working on a document *Al-Mizan: A Covenant for the Earth*, which will be released in October 2021 (UN 2021) and circulate among thousands of mosques worldwide to initiate a programme of lifelong learning for just transitions.

### Faith communities’ transformative learning potential

Faith communities provide all of the five types of enablers of transformative and transgressive, community-based learning highlighted by Didham et al. (2017). First, they provide *belonging* and a space for reflexive and mutual engagement. Second, they provide a shared sense of purpose (*commonality*) – worship of God – within which reflective practices are situated. Third, they provide a *situated* space, where people critically reflect on how they can be agents of just transitions within their own context. Examples of this are a diocese in the North of England establishing its decarbonisation plan for all its institutions, or another diocese in Amazonia trying to respond to land dispossession and water contamination caused by multinational companies. Fourth, faith communities provide an *infrastructure* in terms of a global network of exchange and opportunities for lifelong learning. For example, during the 40 days preceding Easter 2021, the Global Catholic Climate Movement organised a weekly series of adult learning sessions on “Global Healing” with the Catholic Church of England and Wales which was facilitated by the network of dioceses and parishes and schools and drew nearly 1,000 participants (CBCEW 2021). This process of lifelong learning on global healing is also taking place in other denominations and ecumenically, such as the Green Christian movement or Christian Climate Action in the UK (Kidwell 2020).^12^ Finally, faith communities provide *interdependence,* both among the learners of the group and with other communities to which their lives are connected through global economic structures (e.g. beef consumption and people affected by deforestation), and with whom they seek to be in solidarity.

### The critical role of leadership

A key issue, however, which has yet to be analysed, is the critical role of leadership and authority in such transformative, transgressive and community-based learning. Within the Catholic Church, the authority of Pope Francis has been a key factor in facilitating such learning, and has enabled community members to bypass unsupportive leadership at the local level – typically parish priests who see environmental activism as alien to Christian faith and contest the authority of Pope Francis. How structures of authority and leadership are enablers or disablers of education for just transitions remains unexplored in the social movement learning and adult education bodies of literature. Given the crucial role of leadership in social change, this seems to us an urgent agenda of research, and one which HDR 30 does not address either, beyond mention of “the compelling case of young activist Greta Thunberg[’s …] leadership of the Fridays for Future movement” (UNDP 2020, p. 139–140) or generic references to government, civil society and business leadership.

We have argued earlier in this article that HDR 30 missed an opportunity to learn from adult education literature, but it is worth noting that the report is also an example where that literature too could move in a new direction. Achieving just transitions is the greatest challenge facing humanity, yet the field fails to consider the wealth of learning for just transitions already under way in faith communities. There is relatively little in adult education literature about learning across continents, and some of the faith initiatives mentioned above are potentially very significant in this regard. Moreover, learning in faith communities offers new insights into debates regarding the need to get beyond the instrumental and rational, and around the requirement to address relationality.

## Conclusion

While HDR 30 is an inspiring document in many ways, our critical reading of it leads us to the conclusion is that it misses the opportunity to learn from relevant insights available in adult education literature, which is copious. Adult education literature would have suited the purposes of HDR 30 very well, since it agrees with the HDR authors that moving from knowing to doing is far from straightforward. Indeed, its focus on some of the barriers would have been worth the HDR team’s consideration. However, we must also acknowledge the need for adult educators to communicate such messages more effectively to those outside the field.

Unfortunately, HDR 30 builds on an inadequate theory of adult learning and how it contributes to just transitions. The report privileges learning in the market and from the state and internet, but lacks any sense of societal organisations or civil society itself. Moreover, it misses the critical role of faith communities to which most people belong, a weakness it shares with the adult education mainstream. The HDR 30 approach to learning is too individualised, rendering it blind to those structures that support the learning for action it desires and those that obstruct it, issues which have been debated long and hard in adult education over the past 50 years. HDR 30 underplays the role of institutions and collective learning and action, and of culture and place, preferring instead to draw on individualised accounts gleaned from social attitudes surveys, neuroscience and economics.

While Moyer and Sinclair (2020) provide a useful corrective to the downplaying of instrumental learning, HDR 30 fails to find a balance between instrumentalism and transformative learning. It seems to posit a view that is transmissive and where the knowledge is already there and the problems lie in the lack of knowledge and action of individuals. By contrast, there could have been a fruitful exploration of expansive and transgressive learning that is grounded in individuals’, communities’ and institutions’ attempts to build new knowledges and practices for just transitions. As we have demonstrated above, this could usefully have included more on existing learning for just transitions, particularly that undertaken by faith communities.

On a final note, it is worth stating that adult education too has had a blind spot regarding the role of faith communities in transformative and transgressive learning. Given the increasing importance accorded to climate action, and the global spread alongside local grounding of many faith communities, there is a real opportunity for including research on faith-based learning and action for just transitions into mainstream adult education research.

## Footnotes

1 *Big push* theory argues that a massive funding push can trigger a development leap in developing countries (Rosenstein-Rodan 1943).

2 The *Human Development Index* “is a summary measure of average achievement in key dimensions of human development: a long and healthy life, being knowledgeable and have [*sic*] a decent standard of living” (UNDP n.d.). Its purpose is to “emphasize that people and their capabilities should be the ultimate criteria for assessing the development of a country, not economic growth alone” (ibid.).

3 For more information about Transition Network, see https://transitionnetwork.org. The website of Extinction Rebellion is at https://extinctionrebellion.uk [both accessed 2 August 2021].

4 The website of the Via Campesina international peasants’ movement is at https://viacampesina.org/en [accessed 2 August 2021].

5 For more information about Brazil’s Landless Workers Movement, *Movimento dos Trabalhadores Rurais Sem Terra* (MST), see https://www.mstbrazil.org/content/what-mst [accessed 2 August 2021].

6 The online portal of the Eco-Congregation initiative of the Church of England is at http://ecocongregation.org. The Laudato Si’ online Action Platform of the Roman Catholic Church is at https://laudatosiactionplatform.org/ [both accessed 2 August 2021].

7 For more information on UNESCO’s Futures of Education initiative, visit https://en.unesco.org/futuresofeducation [accessed 2 August 2021].

8 The five special issues are “Societal sustainability: The contribution of adult education to sustainable societies” (October 2016); “Non-formal and community learning for sustainable development” (December 2017); “Building sustainable learning cities” (June 2018); “Indigenous knowledges as vital contributions to sustainability” (February 2019); and “Lifelong learning and the Sustainable Development Goals (SDGs): Probing the implications and the effects” (April 2019). For details, see https://link.springer.com/journal/11159/volumes-and-issues.

9 See, for example, the Rainforest Rescue petition “Stop water theft for avocados in Chile!” at https://www.rainforest-rescue.org/petitions/1212/stop-water-theft-for-avocados-in-chile [accessed 3 August 2021].

10 During the annual Fairtraide Fortnight festival in the UK, for example, producers of the Global South visit consumers of the Global North; see https://www.fairtrade.org.uk/Get-Involved/Current-campaigns/Fairtrade-Fortnight/ [accessed 3 August 2021].

11 In 2010, the Pew Research Center’s Forum on Religion & Public Life estimated that 84% of the global population identified with a religious group (PRC 2012), with 15% of the world’s population estimated to be Hindu, 23.2% Muslim and 31.5% Christian (ibid.).

12 Green Christian is online at https://greenchristian.org.ukhttps://christianclimateaction.org/; Christian Climate Action is online at [both accessed 3 August 2021].
